# Prediction of early treatment response to drug-eluting beads chemoembolization for hepatocellular carcinoma using machine learning

**DOI:** 10.3389/fphar.2026.1677843

**Published:** 2026-07-23

**Authors:** Jian Li, Ruopeng Liang, Weijie Wang, Rongtao Zhu, Jiahui Cao, Xiuxian Ma, Yuling Sun

**Affiliations:** 1 Department of Hepatobiliary and Pancreatic Surgery, The First Affiliated Hospital of Zhengzhou University, Zhengzhou, Henan, China; 2 Institute of Hepatobiliary and Pancreatic Diseases, Zhengzhou University, Zhengzhou, Henan, China; 3 Zhengzhou Basic and Clinical Key Laboratory of Hepatopancreatobiliary Diseases, Zhengzhou, Henan, China

**Keywords:** drug-eluting beads chemoembolization, early treatment response, hepatocellular carcinoma, machine learning, predictive value

## Abstract

**Background:**

Drug-eluting bead trans-arterial chemoembolization (DEB-TACE) has been extensively employed as a locoregional therapy for hepatocellular carcinoma (HCC). In this work, machine-learning algorithms are used to forecast early treatment response in HCC patients undergoing DEB-TACE.

**Methods:**

We collected data on patients with HCC who underwent anthracycline-loaded DEB-TACE at our institution over two periods: July 2023 to November 2024 and January to September 2025. The treatment response was evaluated at 1, 3, and 6 months after therapy by dynamic contrast-enhanced computed tomography (CT) based on the modified Response Evaluation Criteria in Solid Tumors (mRECIST). Univariable and multivariable logistic regression analyses were conducted. Independent predictors identified were used to build the predictive model. Machine learning models were evaluated using multiple performance metrics, including AUC, accuracy, sensitivity, and specificity, across the internal validation set and the temporal validation cohort.

**Results:**

A total of 299 HCC patients were included (196 internal cohort, 103 temporal validation cohort), with objective response rates at 1, 3, and 6 months of 56.1%, 51.2%, and 45.9% for the internal cohort and 57.3%, 52.4%, and 49.5% for the temporal validation cohort. Risk factor analysis identified independent predictors of treatment response at various time points, including age, BCLC stage, tumor distribution, and largest tumor diameter. LR showed the highest AUC across all time points, with values of 0.830, 0.840, and 0.854 in the internal validation set and 0.817, 0.832, and 0.839 in the temporal validation cohort at M1, M3, and M6. Subgroup analyses suggested generally consistent predictive performance of the LR model across selected treatment-related subgroups.

**Conclusion:**

In the treatment of hepatocellular carcinoma with DEB-TACE, machine learning models demonstrated promising performance in predicting early treatment response, supporting the development of personalized treatment strategies in the future.

## Introduction

Hepatocellular carcinoma (HCC) is a major global public health burden, with the greatest burden in low- and middle-income countries, especially in East Asia, North Africa, and West Africa (Long et al., 2024; Sung et al., 2021). Based on recent epidemiological statistics, HCC is the sixth most frequently diagnosed cancer and the second leading cause of cancer-related death worldwide, with more than 910,000 new cases and 930,000 deaths occurring each year. China has about 410,000 new cases every year—accounting for 45.3% of the global burden—and over 300,000 deaths, indicating its key position in the global HCC burden (Xie et al., 2023).

Transarterial chemoembolization (TACE) has emerged as the standard of care for intermediate-stage HCC patients (Chang et al., 2020). In conventional TACE, a chemotherapeutic agent—usually admixed with lipiodol—is injected intra-arterially, followed by the delivery of embolic materials to block the tumor-feeding artery, causing ischemic necrosis (Kudo et al., 2020). While conventional TACE has seen widespread adoption, it is compromised by uneven drug delivery, systemic toxicity, and heterogeneous therapeutic response (Ebeling Barbier et al., 2021). In an effort to overcome the shortcomings of conventional TACE, drug-eluting beads TACE (DEB-TACE) was introduced. By utilizing microspheres to deliver agents such as pirarubicin (THP) or epirubicin (EPI) in a sustained and controlled manner, DEB-TACE aims to improve local efficacy while minimizing systemic toxicity (Hai et al., 2024; Liang et al., 2021). In HCC patients treated with DEB-TACE, tumor necrosis and shrinkage usually occur within weeks to months. ORR at 1, 3, and 6 months provides an early, intermediate, and sustained measure of tumor response, helping guide decisions on retreatment or combination therapy. These time points follow international RECIST/mRECIST criteria, and ORR by mRECIST has been shown to correlate with survival outcomes in HCC (Domaratius et al., 2021; Wei and Yang, 2020). While patients with hepatocellular carcinoma undergoing DEB-TACE have been found to exhibit a good early objective response rate (ORR) in general, significant heterogeneity has been reported between time points, patient subsets, and study populations. These inconsistencies point to the pressing need for effective predictive tools to determine probable responders and inform individualized treatment strategy (Cao et al., 2024; Zhou et al., 2018).

Recent studies have shown that various molecular biomarkers and imaging characteristics are associated with the prognosis of HCC. A study utilizing proteomics and transcriptomics analysis found that KIF5B is highly expressed in HCC tumor tissues and correlates with poor prognosis in patients (Qi et al., 2025). Another study used databases such as Kaplan-Meier Plotter to confirm that high expression of miR-3130–5p is associated with poor prognosis in HCC patients (Xu et al., 2024). Researchers also discovered that miR-3130–5p promotes HCC growth by inhibiting the expression of Ferredoxin 1 (FDX1), further linking it to poor patient prognosis (Gudivada and Amajala, 2025). Moreover, galectin-3 has emerged as a promising biomarker for predicting treatment outcomes and prognosis in HCC, with elevated levels associated with worse prognosis (Sotoudeheian, 2024). In addition to these biomarkers, some clinical and imaging characteristics are also correlated with early therapeutic response to DEB-TACE. Well-defined tumor borders, smaller tumor diameter, fewer lesions, and lower alpha-fetoprotein (AFP) and transaminase levels have been significantly associated with increased ORR (Lee et al., 2021; Peng et al., 2020). On the basis of these variables, a number of conventional statistical models have been established to select patients most likely to benefit from treatment. Nevertheless, these models are frequently limited by narrow feature selection, inferior handling of nonlinear relationships, and diminished generalizability, and thus may be inadequate for modeling the heterogeneity of real-world clinical data. With the accelerated development of artificial intelligence (AI), machine learning (ML) has shown superior performance in biomedical and phenotype-based predictive tasks by modeling intricate, nonlinear relationships between high-dimensional data (Liu et al., 2024; Naik et al., 2024). Unlike traditional methods, ML models offer greater flexibility and predictive power by integrating heterogeneous clinical, genetic, imaging, and other multidimensional features (Liu et al., 2023; Wan et al., 2025).

The purpose of this research is to identify clinical, laboratory, and conventional imaging factors associated with early objective response to DEB-TACE at 1, 3, and 6 months in patients with hepatocellular carcinoma. Based on these factors, predictive models were developed using various ML algorithms. Through comparison of model performance, the best-performing algorithm was selected for prediction of early treatment response and was further validated in a temporal split cohort, with the aim of providing a useful tool to assist in personalized therapeutic planning in clinical practice.

## Methods

### Study design and patients

Our research was a single-center study that was carried out after approval had been obtained from the Ethics Review Committee of the First Affiliated Hospital of Zhengzhou University (2022-KY-1519-002). We followed the principles of the Helsinki Declaration, conformed to the Strengthening the Reporting of Observational Studies in Epidemiology (STROBE) guidelines, and met all relevant national laws and regulations. The internal cohort data were retrospectively collected and anonymized, so the ethics committee did not require patient consent. For the prospective validation cohort, all participants provided written informed consent before enrollment.

This study consists of two cohorts, the retrospective (July 2023 to November 2024) and prospective cohorts (January to September 2025). Inclusion criteria: 1) age ≥18 years; 2) diagnosed with primary HCC based on imaging findings and/or histopathology; 3) received DEB-TACE with anthracycline-based chemotherapeutic agents, specifically THP or EPI; 4) complete demographic, medical history, diagnostic, clinical, pathological, treatment, and evaluation data were available; 5) at least 1 month after treatment, contrast-enhanced CT or MRI scan results can be obtained for efficacy evaluation based on mRECIST criteria. Exclusion criteria: 1) history of liver transplantation or any other malignancy; 2) incomplete treatment due to interruption or technical failure of the DEB-TACE procedure; 3) concurrent or previous malignancies other than HCC (including metastatic liver tumors); 4) severe comorbidities or clinical conditions precluding DEB-TACE or post-treatment imaging evaluation (e.g., severe cardiopulmonary or renal dysfunction); 5) missing key baseline variables required for analysis or lack of evaluable follow-up imaging at the prespecified time points; 6) patients with severe cognitive impairment or those who were pregnant or lactating at the time of treatment.

### Data collection

We extracted three main categories of information from the de-identified patient records: (1) clinical information: age, gender, background of underlying illnesses, hepatitis B virus (HBV) infection, hepatitis C virus (HCV) infection, liver cirrhosis, treatment history, microsphere size, microsphere-loaded drug information (including drug type and dosage), number of DEB-TACE procedures, Eastern Cooperative Oncology Group performance status (ECOG PS), Child-Pugh liver function class, Barcelona Clinic Liver Cancer (BCLC) stage, ascites presence, and adjunctive treatments; (2) laboratory test results: white blood cell count (WBC), red blood cell count (RBC), hemoglobin, platelet count, albumin, globulin, prothrombin time (PT), international normalized ratio (INR), total bilirubin (TBIL), direct bilirubin (DBIL), alanine aminotransferase (ALT), aspartate aminotransferase (AST), carcinoembryonic antigen (CEA), carbohydrate antigen 19-9 (CA19-9), and AFP, where AFP was considered positive when ≥20 ng/mL and negative when <20 ng/mL (Cao et al., 2021; Li et al., 2023; Marrero et al., 2018); (3) conventional imaging variables: tumor distribution (classified as unifocal or multifocal) and the largest tumor diameter (defined as the maximum diameter of the largest tumor lesion).

### DEB-TACE procedures

All DEB-TACE procedures were conducted in the Digital Subtraction Angiography (DSA) suite of our hospital by two interventional radiologists with more than 5 years of clinical experience. EqualSpheres® microspheres (ESM; Suzhou Hengrui Medical Equipment Co., Ltd., Jiangsu, China) with diameters of 100, 250, 400, and 500 μm were used as carriers of chemotherapeutic agents as well as embolic agents. Before the procedure, the microspheres chosen were loaded with chemotherapeutic drugs—pirarubicin or epirubicin—at standard dosages of 20 mg, 30 mg, 40 mg, 50 mg or 60 mg. The drugs were initially prepared as a 20 mg/mL solution, fully mixed with the microspheres through a T-connector, and then a nonionic contrast agent was added to form a suspension. The above mixture was gently shaken every 5 min at room temperature for 15 min to finish drug loading.

During the procedure, femoral artery access was gained and a catheter was placed for hepatic arteriography to determine the tumor-feeding arteries. Superselective catheterization was then carried out with a microcatheter and microwire system. The drug-loaded EqualSpheres® microspheres were injected slowly into the target arteries until near-stasis or absolute stasis of contrast flow was reached, which was considered the embolization endpoint. When one vial of microspheres was not sufficient, additional microspheres of different diameters or a lipiodol emulsion was given as required to increase embolization efficacy. In patients with large HCC tumors, several DEB-TACE procedures were carried out according to post-treatment imaging and liver function tests.

### Early treatment response evaluation

Tumor response to DEB-TACE was assessed at 1 month (M1), 3 months (M3) or 6 months (M6) following treatment according to the mRECIST. Contrast-enhanced CT or MRI was utilized to measure change in arterial-phase enhancement of the lesions. Complete response (CR) was the disappearance of arterial-phase enhancement in all target lesions; partial response (PR) a ≥30% reduction in the sum of diameters of arterial-phase enhancing areas from baseline; progressive disease (PD) a ≥20% increase in the sum of diameters of enhancing areas relative to the nadir value, or the occurrence of any new lesion; and stable disease (SD) any change not sufficient to qualify for PR or PD. ORR was the percentage of patients who achieved CR or PR.

### Statistical analysis

Statistical analysis was conducted using Python 3.12. All variables were grouped based on their type. Continuous variables were stated as mean ± standard deviation and compared across groups using Welch’s t-test. Categorical variables were stated as counts and percentages, and compared using the Chi-square test or Fisher’s exact test, where applicable. A two-sided P-value <0.05 was deemed statistically significant. The internal cohort was first randomly divided into a training set (70%) and an internal validation set (30%) before feature selection, preprocessing, and model development. All feature selection, preprocessing, and hyperparameter tuning procedures were performed strictly within the training set. Continuous and categorical variables were preprocessed using Z-score normalization and one-hot encoding, respectively, with all preprocessing parameters fitted only on the training set and then applied unchanged to the internal validation set and temporal validation cohort. No missing values were present in the dataset. Missing outcome data for M3 and M6 due to loss to follow-up were not imputed, and analyses were conducted on available cases at each time point. Univariable and multivariable binary logistic regression analyses were performed at each time point (M1, M3, and M6) to identify variables associated with treatment response. Variables with P < 0.05 in the univariable analyses were subsequently entered into the multivariable models. Model diagnostics were assessed by total sample size (n), number of responders and non-responders, number of variables entered (k), events per variable (EPV = min (events, non-events)/k), and Pseudo R-squared (McFadden). Multicollinearity was evaluated using variance inflation factor (VIF >10 excluded), and sparse-data checks were performed for categorical predictors. Regression coefficients (Coef), odds ratios (OR), and 95% confidence intervals (CI) were estimated for each variable. We performed a post hoc power analysis to assess the statistical power for detecting the observed AUCs.

Using the selected predictors, machine learning models were developed with different algorithms, including random forest (RF), support vector machine (SVM), XGBoost, and logistic regression (LR). Hyperparameter tuning was conducted only in the training set using grid search with five-fold cross-validation. Regularization and early stopping were applied where appropriate to prevent overfitting. Class weights or scale_pos_weight were applied appropriately in different models to address class imbalance. The fitted modeling pipeline was subsequently applied to the internal validation set and the temporal validation cohort for performance evaluation. A temporal validation approach was used in this study, using a prospective temporal validation cohort. Evaluation metrics included the area under the receiver operating characteristic curve (AUC), accuracy, sensitivity, specificity, positive predictive value (PPV), negative predictive value (NPV), and F1 score. We performed the DeLong test to compare AUCs between models. Calibration was evaluated using the Brier score, calibration intercept, and calibration slope. We performed subgroup analysis to evaluate the predictive performance of the best-performing model across drug-loaded and reoperation categories.

## Results

### Participant characteristics

**FIGURE 1:**
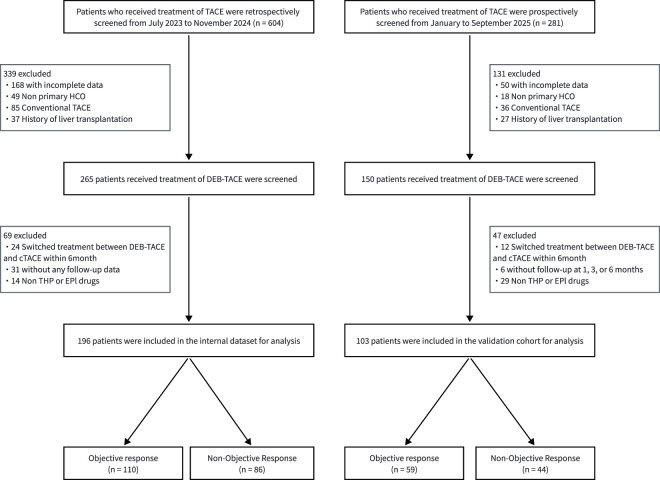
Flow diagram depicting the participant selection process.

In the internal cohort, 604 patients were initially screened, of whom 339 were excluded due to incomplete data, non-primary HCC, conventional TACE, or history of liver transplantation, and an additional 69 were excluded after DEB-TACE for switched treatment, missing ORR follow-up, or use of non-THP/EPI drugs, resulting in 196 patients included; in the temporal cohort, 281 patients were initially screened, 131 were excluded for the same initial reasons, and 47 were further excluded after DEB-TACE, leaving 103 patients included (Figure 1). In the internal cohort, compared with the non-response group, patients in the response group were younger and had a higher proportion of unifocal tumors, smaller largest tumor diameter, better ECOG performance status, better Child–Pugh class, lower platelet counts, a lower proportion of AFP ≥20 ng/mL, and a higher likelihood of receiving epirubicin rather than pirarubicin. In the temporal validation cohort, significant differences were observed in age, tumor distribution, largest tumor diameter, ascites, ECOG performance status, BCLC stage, white blood cell count, albumin, total bilirubin, and direct bilirubin between the response and non-response groups. Table 1 summarizes the baseline characteristics of patients in two cohorts.

**TABLE 1:** Comparison of clinical characteristics of patients in the internal and validation cohorts.

​	Internal cohort			Validation cohort		
Characteristic	CR + PR	SD + PD	*p* - value	CR + PR	SD + PD	*p* - value
Patient	110	86	​	59	44	​
Age (years)	57.5 ± 11.1	62.4 ± 8.2	<0.001	55.2 ± 8.4	60.6 ± 9.1	0.002
Gender	​	​	0.598	​	​	0.588
Male	85 (77.3%)	70 (81.4%)	​	36 (61.0%)	30 (68.2%)	​
Female	25 (22.7%)	16 (19.6%)	​	23 (39.0%)	14 (31.8%)	​
Hypertension	30 (27.3%)	26 (30.2%)	0.767	18 (30.5%)	13 (29.5%)	0.955
Diabetes	11 (10.0%)	9 (10.5%)	1.000	5 (8.5%)	2 (4.5%)	0.696
Coronary heart disease	7 (6.4%)	6 (7.0%)	1.000	2 (3.4%)	2 (4.5%)	0.934
Stroke	2 (1.8%)	6 (7.0%)	0.148	2 (3.4%)	4 (9.1%)	0.398
HB	72 (65.5%)	58 (67.4%)	0.889	39 (66.1%)	28 (63.6%)	0.960
HC	5 (5.8%)	8 (7.3%)	0.906	3 (5.1%)	1 (2.3%)	0.634
Cirrhosis	14 (12.7%)	14 (16.3%)	0.617	11 (18.6%)	9 (20.5%)	0.943
Tumor distribution	​	​	<0.001	​	​	<0.001
Unifocal	65 (59.1%)	26 (30.2%)	​	33 (55.9%)	7 (15.9%)	​
Multifocal	45 (40.9%)	60 (69.8%)	​	26 (44.1%)	37 (84.1%)	​
Largest tumor diameter (cm)	7.9 ± 3.5	9.1 ± 3.4	0.014	7.9 ± 1.8	9.3 ± 1.6	<0.001
Ascites	25 (22.7%)	32 (37.2%)	0.039	11 (18.6%)	31 (70.5%)	<0.001
ECOG PS	​	​	0.006	​	​	<0.001
0	92 (83.6%)	56 (65.1%)	​	48 (81.4%)	20 (45.5%)	​
1	18 (16.4%)	28 (32.6%)	​	9 (15.3%)	14 (31.8%)	​
2	0 (0.0%)	2 (2.3%)	​	2 (3.4%)	10 (22.7%)	​
Child-pugh class	​	​	0.005	​	​	0.140
A	106 (96.4%)	72 (83.7%)	​	52 (88.1%)	33 (75.0%)	​
B	4 (3.6%)	14 (16.3%)	​	7 (11.9%)	11 (25.0%)	​
BCLC stage	​	​	0.410	​	​	<0.001
A	33 (30.0%)	32 (37.2%)	​	40 (67.8%)	6 (13.6%)	​
B	68 (61.8%)	45 (52.3%)	​	18 (30.5%)	19 (43.2%)	​
C	9 (8.2%)	9 (10.5%)	​	1 (1.7%)	19 (43.2%)	​
WBC (×10^9^ cell/L)	5.8 ± 2.7	5.8 ± 2.9	0.975	5.1 ± 1.9	6.2 ± 1.7	0.003
RBC (×10^12^ cell/L)	4.1 ± 0.7	4.1 ± 0.6	0.461	4.2 ± 0.4	4.1 ± 0.5	0.120
Hemoglobin	123.6 ± 21.4	125.3 ± 20.3	0.576	127.1 ± 15.1	123.1 ± 15.4	0.197
Platelet	164.2 ± 80.6	190.9 ± 88.4	0.029	162.1 ± 46.2	165.6 ± 54.5	0.733
Albumin	37.6 ± 7.2	37.9 ± 7.2	0.750	40.74 ± 4.6	36.2 ± 4.5	0.009
Globulin	31.8 ± 5.9	30.4 ± 6.4	0.106	30.9 ± 3.4	31.0 ± 4.9	0.839
Prothrombin time (s)	12.8 ± 1.5	12.5 ± 1.7	0.157	12.2 ± 0.8	12.2 ± 1.1	0.721
INR	1.4 ± 0.9	1.2 ± 1.3	0.285	1.3 ± 0.5	1.2 ± 0.8	0.901
TBIL (µmol/L)	15.6 ± 9.5	14.7 ± 8.8	0.503	12.0 ± 3.4	13.6 ± 4.2	0.039
DBIL (µmol/L)	7.8 ± 5.4	7.5 ± 5.4	0.701	5.8 ± 1.7	6.6 ± 1.9	0.029
ALT (U/L)	53.2 ± 60.7	49.8 ± 57.3	0.693	57.4 ± 34.1	59.0 ± 40.5	0.415
AST (U/L)	70.8 ± 74.6	58.1 ± 54.5	0.173	71.0 ± 40.6	73.7 ± 48.7	0.761
AFP (ng/mL)	​	​	0.048	​	​	0.389
<20	51 (46.4%)	27 (31.4%)	​	29 (49.2%)	17 (38.6%)	​
≥20	59 (53.6%)	59 (68.6%)	​	30 (50.8%)	27 (61.4%)	​
CEA (ng/mL)	2.6 ± 1.6	3.1 ± 2.3	0.237	2.6 ± 1.1	2.9 ± 1.4	0.160
CA19-9 (U/mL)	23.3 (11.5, 37.3)	20.9 (10.6, 38.2)	0.703	20.3 (14.8, 26.8)	19.1 (13.1, 29.7)	0.409
ESM diameter	​	​	0.813	​	​	0.071
100–300 µm	72 (65.5%)	54 (62.8%)	​	41 (69.5%)	22 (50.0%)	​
300–500 µm	38 (34.5%)	32 (37.2%)	​	18 (30.5%)	22 (50.0%)	​
Drug-loaded	​	​	0.004	​	​	0.361
Pirarubicin	45 (40.9%)	54 (62.8%)	​	41 (69.5%)	22 (50.0%)	​
Epirubicin	65 (59.1%)	32 (37.2%)	​	18 (30.5%)	22 (50.0%)	​
Drug-loaded dose			0.002	​	​	0.045
<40 mg	28 (25.5%)	41 (47.7%)	​	20 (33.9%)	19 (43.2%)	​
≥40 mg	82 (74.5%)	45 (52.3%)	​	39 (66.1%)	25 (56.8%)	​
Treatment frequency	​	​	0.391	​	​	0.376
Single-session	80 (72.7%)	68 (79.1%)	​	41 (69.5%)	26 (59.1%)	​
Multiple-session	30 (27.3%)	18 (20.9%)	​	18 (30.5%)	18 (40.9%)	​
Adjunctive therapeutic	80 (72.7%)	64 (74.4%)	0.289	45 (76.3%)	36 (81.8%)	0.911
Previous treatments	​	​	​	​	​	​
cTACE	27 (24.5%)	14 (16.3%)	0.217	11 (18.6%)	8 (18.2%)	1.000
Surgery	7 (6.4%)	14 (16.3%)	0.046	10 (16.9%)	6 (13.6%)	0.853
Chemotherapy	1 (0.9%)	2 (2.3%)	0.829	1 (1.7%)	3 (6.8%)	0.633
Radiofrequency ablation	4 (3.6%)	3 (3.5%)	1.000	1 (1.7%)	0 (0.0%)	1.000
Immunotherapy + targeted	33 (30.0%)	25 (29.1%)	1.000	18 (30.5%)	22 (50.0%)	0.071

CR, complete response; PR, partial response; SD, stable disease; PD, progressive disease; HB, hepatitis B; HC, hepatitis C; ECOG PS, eastern cooperative oncology group performance status; BCLC, barcelona clinic liver cancer; WBC, white blood cell; RBC, red blood cell; INR, international normalized ratio; TBIL, total bilirubin; DBIL, direct bilirubin; ALT, alanine aminotransferase; AST, aspartate aminotransferase; AFP, alpha fetoprotein.

### Early treatment response

**FIGURE 2:**
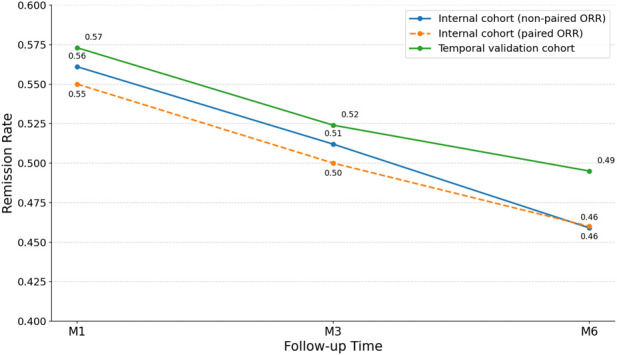
Trend of objective response rate at 1, 3, and 6 months in the internal and temporal validation cohort.

Treatment response was evaluated according to mRECIST in all patients in the internal cohort at 1 month after treatment, with follow-up assessments available for 166 patients (84.7%) at 3 months and 133 patients (67.9%) at 6 months. The ORR was 56.1% (110/196) at 1 month, 51.2% (85/166) at 3 months, and 45.9% (61/133) at 6 months. In the temporal validation cohort, all 103 patients had available response assessments at 1, 3, and 6 months, with ORRs of 57.3% (59/103), 52.4% (54/103), and 49.5% (51/103), respectively. Figure 2 shows the ORR trend over time, demonstrating a gradual decline in both the internal cohort and the temporal validation cohort.

### Independent risk factors for early treatment response

Univariable and multivariable logistic regression analyses were performed at each follow-up time point (M1, M3, and M6) to identify independent risk factors at each time point, as shown in Table 2. At M1, independent predictors of objective response were age (OR 0.94, 95% CI 0.90–0.97; *p* < 0.001), tumor distribution (OR 0.28, 95% CI 0.15–0.55; *p* < 0.001), ascites (OR 0.31, 95% CI 0.14–0.67; *p* = 0.003), platelet count (OR 0.99, 95% CI 0.98–1.00; *p* = 0.013), and globulin level (OR 1.11, 95% CI 1.04–1.16; *p* = 0.002). At M3, significant predictors included hypertension (OR 1.03, 95% CI 1.01–1.05; *p* = 0.009), ECOG PS (OR 0.72, 95% CI 0.62–0.84; *p* < 0.001), largest tumor diameter (OR 0.76, 95% CI 0.67–0.87; *p* < 0.001), and platelet count (OR 1.01, 95% CI 1.00–1.02; *p* = 0.005). At M6, independent predictors were platelet count (OR 1.01, 95% CI 1.00–1.02; *p* = 0.034), BCLC stage (OR 2.54, 95% CI 1.02–6.33; *p* = 0.045), largest tumor diameter (OR 0.60, 95% CI 0.48–0.76; *p* < 0.001), drug-loaded (OR 2.75, 95% CI 1.06–7.14; *p* = 0.038), and ECOG PS (OR 0.11, 95% CI 0.03–0.37; *p* < 0.001). These results indicate that tumor characteristics and patient clinical status dynamically affect treatment response over time. Supplementary Table S1 provides additional model diagnostics, including sample size, number of variables entered, and events-per-variable (EPV) ratios, which support the overall interpretability of the models.

**TABLE 2:** Independent risk factors analyses for objective response based on mRECIST.

Variable	Coef	OR	95% CI	VIF	*p* - value
M1					
Age	−0.067	0.94	0.90–0.97	1.06	<0.001
Tumor_distribution	−1.259	0.28	0.15–0.55	1.04	<0.001
Ascites	−1.173	0.31	0.14–0.67	1.11	0.003
Platelet	−0.005	0.99	0.98–1.00	1.01	0.013
Globulin	0.093	1.11	1.04–1.16	1.04	0.002
M3					
Hypertension	0.028	1.03	1.01–1.05	1.02	0.009
ECOG PS	−0.326	0.72	0.62–0.84	1.08	<0.001
Largest tumor diameter	−0.272	0.76	0.67–0.87	1.09	<0.001
Platelet	0.008	1.01	1.00–1.02	1.01	0.005
M6					
Platelet	0.006	1.01	1.00–1.02	1.01	0.034
BCLC	0.932	2.54	1.02–6.33	1.89	0.045
Largest tumor diameter	−0.507	0.60	0.48–0.76	2.00	<0.001
Drug-loaded	1.012	2.75	1.06–7.14	1.07	0.038
ECOG PS	−2.209	0.11	0.03–0.37	1.14	<0.001

OR, odds ratio; CI, confidence interval; VIF, variance inflation factor; BCLC, barcelona clinic liver cancer; ECOG PS, eastern cooperative oncology group performance status; RBC, red blood cell; AFP, alpha fetoprotein.

### Comparison of machine learning based prediction models

**FIGURE 3:**
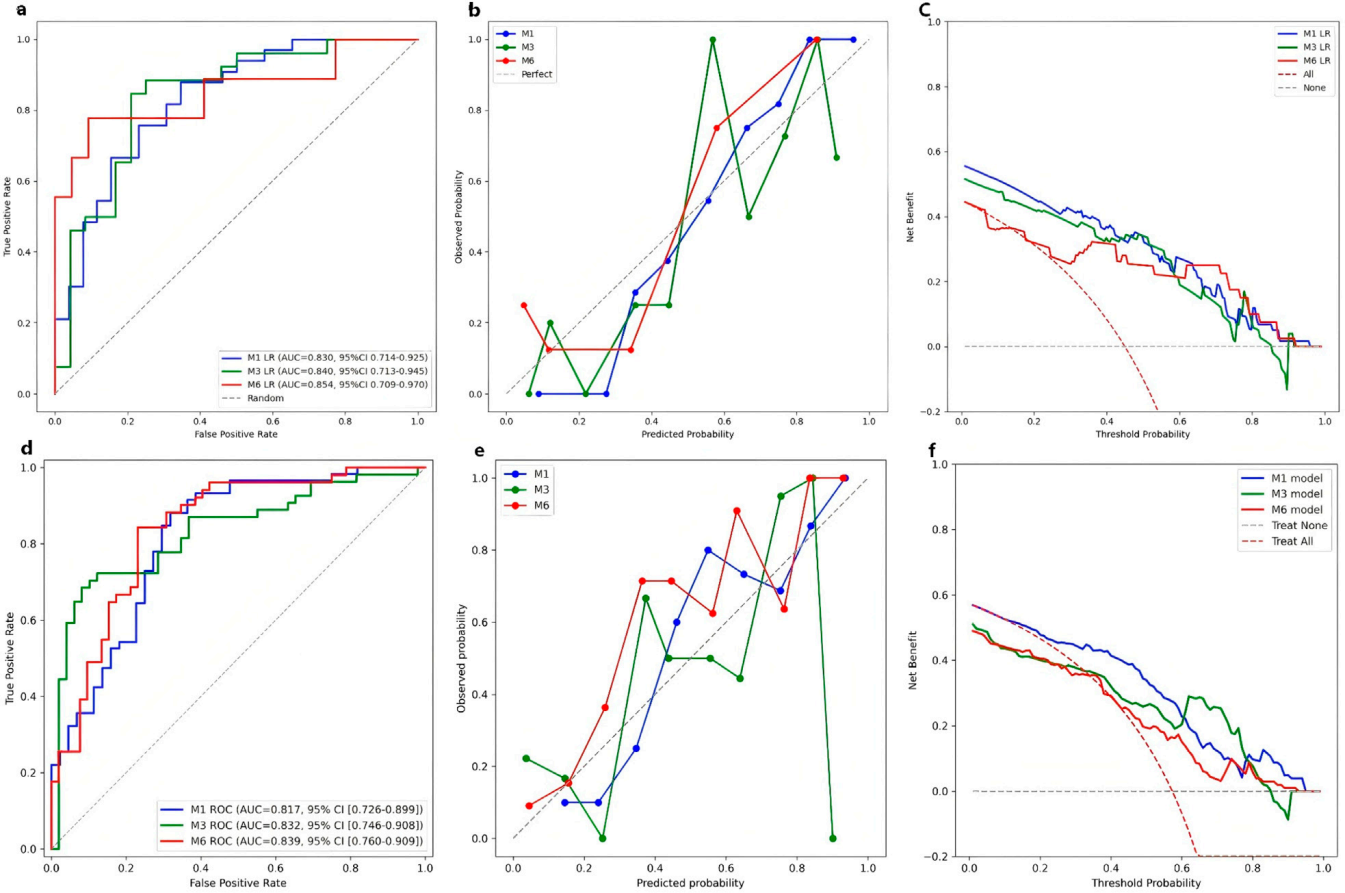
Performance of the logistic regression model at different follow-up times (1, 3, and 6 months) in the internal validation set and temporal validation cohort. **(a–c)** Internal validation set: **(a)** ROC curves, **(b)** calibration curves, **(c)** decision curve analysis; **(d–f)** Temporal validation cohort: **(d)** ROC curves, **(e)** calibration curves, **(f)** decision curve analysis.

The predictive performance of the machine learning models was evaluated at M1, M3, and M6 in the internal validation set and tested in the temporal validation cohort (Table 3). Across all time points, LR model achieved the highest discrimination, with AUCs of 0.830, 0.840, and 0.854 at M1, M3, and M6 in the internal validation set, respectively, and 0.817, 0.832, and 0.839 in the temporal validation cohort (Figure 3). LR also demonstrated generally acceptable calibration, as reflected by Brier scores (0.173–0.149 in the internal validation set; 0.168–0.179 in the temporal cohort) and the calibration plots, although some instability in calibration intercepts and slopes was observed across time points. Accuracy, sensitivity, specificity, PPV, NPV, and F1 scores also suggested generally favorable performance for LR across follow-up time points. Other models including random forest, SVM, and XGBoost showed comparable trends but generally lower AUC and slightly inferior calibration. The DeLong test results show that at the M1 and M6 time points, LR exhibits significant differences in AUC compared with some other models, whereas no significant differences are observed among models at the M3 time point (Supplementary Table S2). Power analysis results suggested adequate statistical power for detecting the observed AUCs at each follow-up time point, as summarized in Supplementary Table S4.

**TABLE 3:** The predictive performance of multiple machine learning models at different follow-up times.

Metrics	RF	XGBoost	SVM	LR
M1				
Internal validation set (n = 59)				
AUC (95% CI)	0.753 (0.623–0.867)	0.721 (0.585–0.874)	0.816 (0.699–0.911)	0.830 (0.714–0.925)
Accuracy	0.644	0.644	0.746	0.763
Sensitivity	0.727	0.667	0.970	0.848
Specificity	0.538	0.615	0.462	0.654
PPV	0.667	0.688	0.696	0.757
NPV	0.609	0.593	0.923	0.773
F1 score	0.696	0.677	0.810	0.800
Brier score	0.196	0.237	0.209	0.173
Calibration intercept	0.070	0.002	−0.049	−0.023
Calibration slope	1.443	0.444	4.718	1.763
Temporal validation cohort (n = 103)				
AUC (95% CI)	0.711 (0.597–0.817)	0.655 (0.542–0.761)	0.771 (0.667–0.868)	0.817 (0.726–0.89)
Accuracy	0.699	0.631	0.757	0.767
Sensitivity	0.780	0.661	0.864	0.814
Specificity	0.591	0.591	0.614	0.705
PPV	0.719	0.684	0.750	0.787
NPV	0.667	0.565	0.771	0.738
F1 score	0.748	0.672	0.803	0.800
Brier score	0.208	0.264	0.179	0.168
Calibration intercept	0.038	0.082	−0.065	0.095
Calibration slope	0.912	0.247	1.072	1.273
M3				
Internal validation set (n = 50)				
AUC (95% CI)	0.777 (0.626–0.916)	0.707 (0.547–0.862)	0.793 (0.651–0.924)	0.840 (0.713–0.945)
Accuracy	0.740	0.660	0.760	0.820
Sensitivity	0.731	0.654	0.846	0.846
Specificity	0.750	0.667	0.667	0.792
PPV	0.760	0.680	0.733	0.815
NPV	0.720	0.640	0.800	0.826
F1 score	0.745	0.667	0.786	0.830
Brier score	0.190	0.235	0.177	0.164
Calibration intercept	0.039	0.046	−0.165	−0.090
Calibration slope	0.833	0.357	0.891	1.082
Temporal validation cohort (n = 103)				
AUC (95% CI)	0.813 (0.725–0.893)	0.758 (0.662–0.849)	0.808 (0.714–0.889)	0.832 (0.746–0.908)
Accuracy	0.738	0.680	0.738	0.738
Sensitivity	0.704	0.648	0.722	0.778
Specificity	0.776	0.714	0.755	0.694
PPV	0.776	0.714	0.765	0.737
NPV	0.704	0.648	0.712	0.739
F1 score	0.738	0.680	0.743	0.757
Brier score	0.182	0.214	0.181	0.179
Calibration intercept	0.377	0.391	0.380	0.429
Calibration slope	0.888	0.525	0.862	0.598
M6				
Internal validation set (n = 40)				
AUC (95% CI)	0.846 (0.770–0.914)	0.838 (0.694–0.955)	0.847 (0.769–0.916)	0.854 (0.709–0.970)
Accuracy	0.728	0.825	0.767	0.800
Sensitivity	0.510	0.667	0.706	0.611
Specificity	0.942	0.955	0.827	0.955
PPV	0.897	0.923	0.800	0.917
NPV	0.662	0.778	0.741	0.750
F1 score	0.650	0.774	0.750	0.733
Brier score	0.178	0.154	0.170	0.149
Calibration intercept	0.518	0.398	0.204	0.423
Calibration slope	1.631	0.838	1.701	0.986
Temporal validation cohort (n = 103)				
AUC (95% CI)	0.835 (0.697–0.953)	0.813 (0.730–0.889)	0.813 (0.659–0.937)	0.839 (0.760–0.909)
Accuracy	0.800	0.709	0.800	0.699
Sensitivity	0.667	0.49	0.722	0.549
Specificity	0.909	0.923	0.864	0.846
PPV	0.857	0.862	0.812	0.778
NPV	0.769	0.649	0.792	0.657
F1 score	0.750	0.625	0.765	0.644
Brier score	0.167	0.201	0.174	0.179
Calibration intercept	0.373	1.093	0.201	0.663
Calibration slope	0.820	0.822	1.326	1.035

RF, random forest; XGBoost, extreme gradient boosting; SVM, support vector machine; LR, logistic regression; AUC, area under receiver operating characteristic curve; PPV, positive predictive value; NPV, negative predictive value.

### SHAP analysis of optimal machine learning model

**FIGURE 4:**
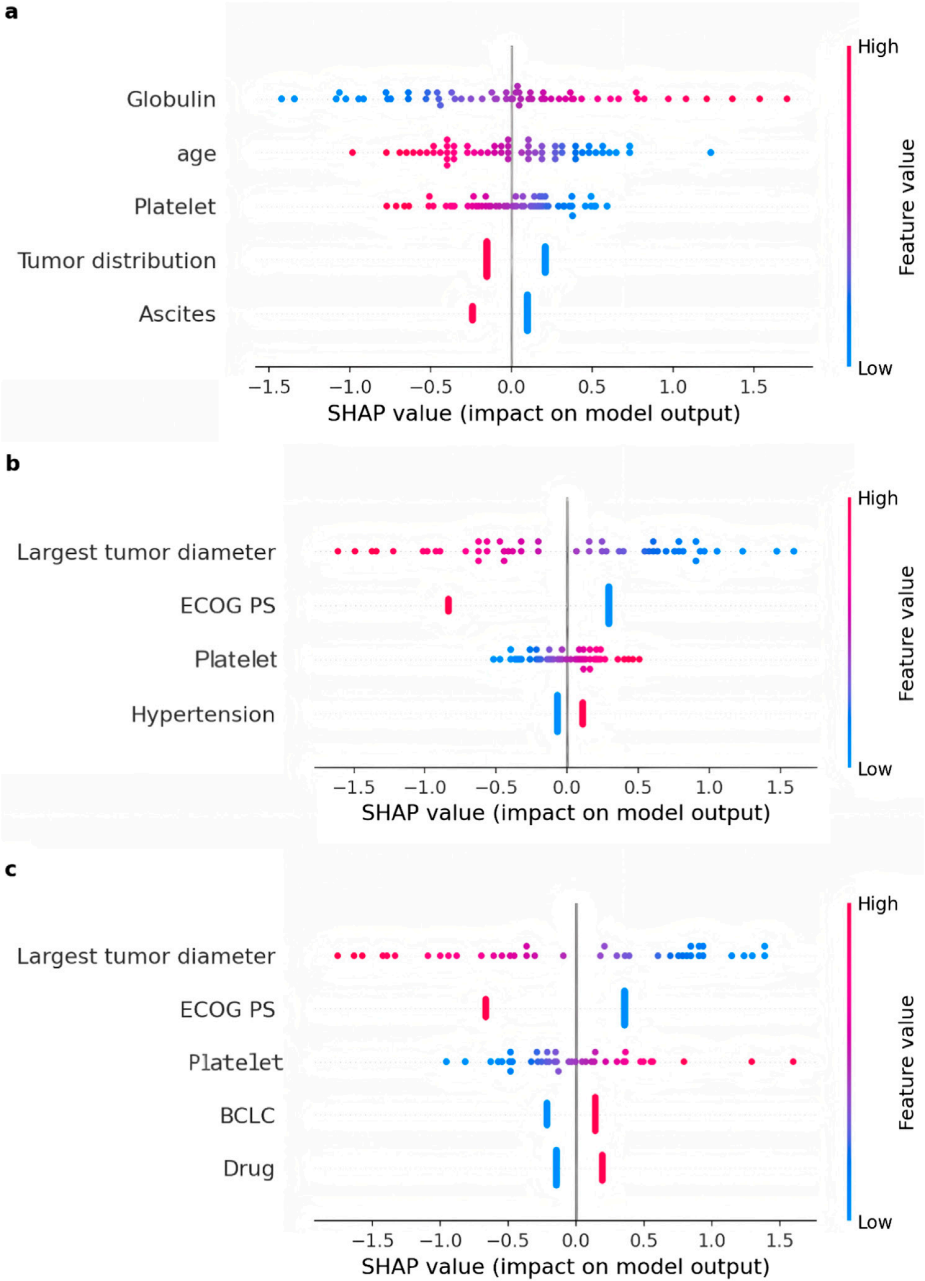
SHAP analysis of the optimal machine learning model. **(a)** Beeswarm plot of the logistic regression model at 1 month; **(b)** Beeswarm plot of the logistic regression model at 3 months; **(c)** Beeswarm plot of the logistic regression model at 6 months. Each point represents an individual SHAP value for a feature in a single patient. The x-axis indicates the impact of the feature on the model output (SHAP value), where positive values increase the predicted risk and negative values decrease it. Color indicates feature value: red = high, blue = low.

Figure 4 shows SHAP analysis results for the optimal machine learning model. Panel a displays the SHAP analysis for the optimal LR model at 1 month, showing the features ranked by impact on treatment response. The top-ranked features were globulin, age, platelet, tumor distribution, and ascites. Panel b displays the SHAP analysis for the model at 3 months, with the top-ranked features being largest tumor diameter, ECOG PS, platelet, and hypertension. Panel c displays the SHAP analysis for the model at 6 months, where the most influential features in descending order of SHAP values were largest tumor diameter, ECOG PS, platelet, BCLC stage, and drug-loaded.

### Subgroup analysis of predictive performance

Subgroup analysis suggested generally consistent discriminative performance across selected treatment-related subgroups in the temporal validation cohort. At M1 and M3, AUCs were comparable between the pirarubicin and epirubicin groups, as well as between the single- and multiple-session reoperation groups. At M6, the reoperation subgroups also showed comparable discrimination (Supplementary Table S5).

## Discussion

This research examined the ORR of HCC patients treated with DEB-TACE within a 1- to 6-month follow-up period, which presented a trend of gradual decrease. We also examined independent risk factors related to treatment response at various follow-up time points and identified that the influencing factors differed at different times. Four prediction models were developed based on the selected important variables, and their performance was evaluated at 1, 3, and 6 months in both the internal validation set and the temporal validation cohort. All models demonstrated promising predictive ability, with performance increasing slightly from 1 to 3–6 months. Among them, the LR model achieved the best performance across all time points, suggesting that a parsimonious and interpretable model may be suitable for preliminary prediction of early treatment response. The DeLong test provided additional comparative evidence, although performance differences varied across time points and algorithms. SHAP analysis indicated that the features with the highest impact on model predictions were globulin at 1 month, largest tumor diameter at 3 months, and largest tumor diameter at 6 months. Subgroup analyses suggested that the model retained generally consistent discriminative ability across treatment-related strata; however, given the small sample sizes in several subgroups, these findings should be interpreted cautiously.

In recent years, increasing efforts have been devoted to identifying biomarkers for predicting early treatment response to TACE, including both DEB-TACE and conventional TACE. In contrast, most prior studies have focused on prediction at a single early endpoint (e.g., 30–60 days or 3 months after TACE), which limits their ability to support iterative decision-making during follow-up. One study reported that irregular tumor margins, increased number of nodules, and lower pretreatment platelet counts were independent predictors of early objective response 30–60 days following TACE. A combined predictive model based on the above variables yielded an AUC of 0.755, reflecting good discriminatory power (Li et al., 2022). Another multicenter retrospective research established a nomogram based on AFP levels, PVTT, tumor number, and largest tumor diameter to predict early objective response 3 months after TACE. The model had good performance in both the training and validation sets, with C-indices of 0.853 and 0.800, respectively (Li et al., 2023). Besides, researchers indicated that 1 month after DEB-TACE treatment, HCC patients had a CR rate of 47.6% and a PR rate of 29.1%; a preoperative CT texture feature-based predictive model showed good performance, with an AUC of 0.733 (Tipaldi et al., 2021). However, these traditional statistical models were typically limited to a single early time point and did not fully leverage longitudinal follow-up data. In this study, we developed and evaluated a dynamic, multi-timepoint predictive framework using multiple machine learning algorithms at 1, 3, and 6 months after DEB-TACE, systematically comparing the predictive factors and the optimal algorithm across different time points. This approach enables the integration of information from multiple follow-up times and captures more complex relationships among clinical variables.

Relative to conventional statistical techniques, ML models were more capable of extracting complex and nonlinear relationships between multidimensional clinical and imaging variables (Vithayathil et al., 2025). A number of studies showed that ML-based predictive models had high accuracy when combining multiparametric imaging data (Dong et al., 2021; Lu et al., 2025; Shi et al., 2024). Further, Zhang et al. demonstrated that a model built using clinical and CT imaging features achieved better predictive performance for objective response to DEB-TACE than a model using clinical features alone (Zhang et al., 2024). Likewise, He et al. constructed a random forest model with CT texture features and conventional imaging variables, with an AUC of 0.947 and predictive accuracy of 89.5% for treatment response to TACE (An et al., 2023). Our findings are generally consistent with previous studies and indicate that the machine learning models achieved promising discrimination across multiple follow-up time points (M1, M3, and M6), with AUC values tending to increase from 1 to 6 months. Among these models, LR demonstrated promising performance, suggesting that a parsimonious and interpretable model may be suitable for preliminary prediction tasks based on routine clinical and conventional imaging variables.

Interestingly, our study noted a trend of decreasing ORR over time, which suggests a possible association with the mechanisms of tumor recurrence, lesion progression, and DEB-TACE local control in certain patients, among other factors (Villaruz and Socinski, 2013). Furthermore, the administration of varying chemotherapeutic agents between patients—THP vs. EPI, for example—may have been a factor in variability of treatment durability, as previous studies have suggested that variations in drug-release kinetics and cytotoxicity can impact durable tumor control and, in turn, ORR at later follow-up time points (Zhu et al., 2024). Alternatively, loss to follow-up was greater over time, and patients with favorable early responses might have been less inclined to return, leading to a follow-up cohort with a bias toward worse outcomes. However, all analyses were based on data from patients who completed follow-up at each time point, minimizing the impact of missing data on the overall results.

Additionally, the independent risk factors varied across the three follow-up time points, reflecting the dynamic nature of patient physiology and tumor biology. At 1 month (M1), age was the primary predictor, suggesting that elderly patients with decreased liver reserve and compromised immune function experienced poorer early responses (Yang et al., 2018; Zhou, 2018). At the M3 and M6 time point, ECOG PS and largest tumor diameter were identified as key predictors. ECOG performance status and largest tumor diameter became the most influential factors. ECOG PS indicates overall functional reserve, with lower scores limiting treatment tolerance, while larger tumor diameter reflects higher tumor burden, impacting longer-term outcomes (Forner et al., 2018; Llovet et al., 2002). The association between largest tumor diameter and treatment response suggests that tumor burden may contribute to longer-term outcomes after DEB-TACE (Forner et al., 2018; Llovet et al., 2002; Guo et al., 2021; Lin et al., 2024). Platelet count also showed time-dependent effects, highlighting how evolving patient physiology and tumor progression influence DEB-TACE efficacy. Consistent with these findings, SHAP analysis demonstrated that the influence of key predictors on treatment response changed over time. Tumor burden, ECOG PS, and platelet count remained consistently important, highlighting actionable insights such as closer monitoring for patients with poor ECOG PS or large tumors. Clinically, the predicted probabilities, together with the time-dependent importance of key predictors, may help identify patients with a low likelihood of objective response after DEB-TACE and support closer imaging surveillance, earlier retreatment evaluation, or consideration of combined therapeutic strategies.

There are a number of limitations to this study. First, this was a single-center study with a relatively limited sample size, and as such, the generalizability of the results may be limited. Second, as the study included a retrospective cohort, some outcome follow-up data were missing, and we cannot completely rule out selection and information bias. Third, although clinical, laboratory, and conventional imaging variables were considered, the imaging information was limited to tumor distribution and largest tumor diameter. More detailed imaging features and radiomics-derived features should be explored in future studies. Fourth, although we conducted validation using a prospective temporal cohort, this validation was performed within the same center, and the generalizability to populations from different regions remains untested. Finally, the relatively short follow-up duration and the increasing proportion of lost-to-follow-up patients may have introduced bias, and long-term endpoints such as progression-free survival or overall survival were not assessed. Therefore, larger multicenter studies with external validation, incorporating radiomics, genomics, and long-term outcomes such as OS and PFS, are essential to further evaluate the reproducibility, generalizability, and clinical relevance of our findings.

## Conclusion

ML models demonstrated promising predictive performance by leveraging dynamic clinical and tumor-related variables across multiple follow-up time points, and were evaluated in a prospective temporal validation cohort. These findings support the potential utility of such models for personalized treatment planning and informed clinical decision-making.

## Data Availability

The original contributions presented in the study are included in the article/Supplementary Material, further inquiries can be directed to the corresponding author.
