# Supplementary material for: Prediction of early treatment response to drug-eluting beads chemoembolization for hepatocellular carcinoma using machine learning
Source: Front Pharmacol. 2026 Jul 23;17:1677843. doi: 10.3389/fphar.2026.1677843 (PMC13443040; doi:10.3389/fphar.2026.1677843)
Supplement: Supplementary file 1 [file Table1.docx]

**Table S1. Diagnostics for multivariable logistic regression models used to identify independent risk factors at 1, 3, and 6 months**

| Metric | M1 | M3 | M6 |
| --- | --- | --- | --- |
| Total sample size (n) | 137 | 116 | 93 |
| Responders | 77 | 59 | 43 |
| Non-responders | 60 | 57 | 50 |
| Variables entered into multivariable model (k) | 10 | 7 | 5 |
| EPV = min(events, non-events) / k | 6 | 8.1 | 8.6 |
| Pseudo R-squared (McFadden) | 0.317 | 0.359 | 0.453 |
| sparse-data check | a | b | b |
| a, no evidence of sparse-data bias; b, sparse-data bias potentially present for ECOG PS (1 zero cell observed). | | | |
