# Supplementary material for: Prediction of early treatment response to drug-eluting beads chemoembolization for hepatocellular carcinoma using machine learning
Source: Front Pharmacol. 2026 Jul 23;17:1677843. doi: 10.3389/fphar.2026.1677843 (PMC13443040; doi:10.3389/fphar.2026.1677843)
Supplement: Supplementary file 2 [file Table2.docx]

**Table S2. Comparison of model AUCs across different machine learning algorithms using the DeLong test at each time point.**

| Comparison | P - Value | P - Value |
| --- | --- | --- |
|  | Temporal validation cohort | Internal validation set |
| M1 |  |  |
| LR vs RF | 0.012 | 0.001 |
| LR vs SVM | 0.476 | 0.006 |
| LR vs XGB | 0.003 | 0.001 |
| RF vs SVM | 0.024 | 0.098 |
| RF vs XGB | 0.149 | 0.785 |
| SVM vs XGB | 0.001 | 0.069 |
| M3 |  |  |
| LR vs RF | 0.295 | 0.252 |
| LR vs SVM | 0.239 | 0.171 |
| LR vs XGB | 0.067 | 0.102 |
| RF vs SVM | 0.852 | 0.749 |
| RF vs XGB | 0.277 | 0.488 |
| SVM vs XGB | 0.361 | 0.648 |
| M6 |  |  |
| LR vs RF | 0.005 | 0.346 |
| LR vs SVM | 0.003 | 0.598 |
| LR vs XGB | 0.002 | 0.598 |
| RF vs SVM | 0.355 | 0.578 |
| RF vs XGB | 0.737 | 0.578 |
| SVM vs XGB | 0.432 | 0.909 |
| M1, 1 month; M3, 3 months; M6, 6 months; RF, random forest; XGBoost, extreme gradient boosting; SVM, support vector machine; LR, logistic regression. | | |
