# Supplementary material for: Prediction of early treatment response to drug-eluting beads chemoembolization for hepatocellular carcinoma using machine learning
Source: Front Pharmacol. 2026 Jul 23;17:1677843. doi: 10.3389/fphar.2026.1677843 (PMC13443040; doi:10.3389/fphar.2026.1677843)
Supplement: Supplementary file 3 [file Table3.docx]

**Table S3. Final hyperparameters adopted in the four machine learning models**

| Model | Parameter | Search space | Final value |
| --- | --- | --- | --- |
| RF | n_estimators | 50, 100, 200 | 100 |
|  | max_depth | 10, 20, 30 | 20 |
|  | min_samples_split | 2, 5 | 2 |
|  | min_samples_leaf | 1, 2 | 1 |
|  | criterion | gini, entropy | gini |
|  | class_weight | balanced | balanced |
| LR | C | 0.01, 0.1, 1, 10 | 1 |
|  | penalty | l2 | l2 |
|  | solver | lbfgs | lbfgs |
|  | class_weight | balanced | balanced |
| SVM | kernel | linear, rbf | rbf |
|  | C | 0.1, 1, 10, 100 | 1 |
|  | gamma | scale, 0.01, 0.03, 0.1 (RBF only) | scale |
|  | class_weight | balanced | balanced |
| XGBoost | learning_rate | 0.01, 0.1, 0.2 | 0.1 |
|  | n_estimators | 50, 100, 200 | 100 |
|  | max_depth | 3, 6, 9 | 6 |
|  | subsample | 0.8, 0.9, 1.0 | 0.9 |
|  | scale_pos_weight | balanced | 1 (adjusted for imbalance) |
| RF, random forest; XGBoost, extreme gradient boosting; SVM, support vector machine; LR, logistic regression.  **Notes:** All models use 5-fold cross-validation (Stratified) and seed = 42. Hyperparameters were selected via grid search in the training set. Regularization and early stopping were applied where appropriate to prevent overfitting. Class imbalance handling: RF, LR, SVM use class_weight = 'balanced'; XGBoost uses scale_pos_weight. | | | |
