# Supplementary material for: Prediction of early treatment response to drug-eluting beads chemoembolization for hepatocellular carcinoma using machine learning
Source: Front Pharmacol. 2026 Jul 23;17:1677843. doi: 10.3389/fphar.2026.1677843 (PMC13443040; doi:10.3389/fphar.2026.1677843)
Supplement: Supplementary file 4 [file Table4.docx]

**Table S4. Power analysis and sample characteristics for logistic regression models at each follow-up time point.**

| **Cohort** | **AUC** | **Total** | **Events** | **Non-events** | **Power** |
| --- | --- | --- | --- | --- | --- |
| M1_internal validation | 0.830 | 59 | 33 | 26 | 1 |
| M3_internal validation | 0.840 | 50 | 26 | 24 | 1 |
| M6_internal validation | 0.854 | 40 | 18 | 22 | 1 |
| M1_temporal validation | 0.817 | 103 | 59 | 44 | 1 |
| M3_temporal validation | 0.832 | 103 | 54 | 49 | 1 |
| M6_temporal validation | 0.839 | 103 | 51 | 52 | 1 |
| AUC, area under receiver operating characteristic curve; M1, 1 month; M3, 3 months; M6, 6 months. | | | | | |
