# Supplementary material for: Prediction of early treatment response to drug-eluting beads chemoembolization for hepatocellular carcinoma using machine learning
Source: Front Pharmacol. 2026 Jul 23;17:1677843. doi: 10.3389/fphar.2026.1677843 (PMC13443040; doi:10.3389/fphar.2026.1677843)
Supplement: Supplementary file 5 [file Table5.docx]

**Table S5. Subgroup analysis of logistic regression model predictive performance by drug type and reoperation status in the temporal validation cohort.**

|  | **Subgroup** | **Group** | **n** | **AUC** | **95% CI** |
| --- | --- | --- | --- | --- | --- |
| M1 | Drug_loaded | Pirarubicin | 63 | 0.818 | 0.689-0.925 |
|  |  | Epirubicin | 40 | 0.806 | 0.663-0.926 |
|  | Reoperation | Single-session | 67 | 0.797 | 0.672-0.900 |
|  |  | Multiple-session | 36 | 0.84 | 0.682-0.960 |
| M3 | Drug_loaded | Pirarubicin | 58 | 0.82 | 0.694-0.925 |
|  |  | Epirubicin | 45 | 0.844 | 0.691-0.954 |
|  | Reoperation | Single-session | 67 | 0.793 | 0.672-0.901 |
|  |  | Multiple-session | 36 | 0.883 | 0.741-0.987 |
| M6 | Reoperation | Single-session | 67 | 0.881 | 0.789-0.954 |
|  |  | Multiple-session | 36 | 0.787 | 0.610-0.928 |
| AUC, area under receiver operating characteristic curve; CI, confidence interval.  Drug_loaded was included as a predictor in the logistic regression-M6 model, so subgroup analysis by drug_loaded was not performed to avoid circularity. | | | | | |
